# Localized Giant Cell Tenosynovial Tumor Seen in the Knee Joint

**DOI:** 10.1155/2014/840243

**Published:** 2014-03-04

**Authors:** Ozan Beytemür, Cem Albay, Ümit Seza Tetikkurt, Mehmet Öncü, Mehmet Ali Baran, Sever Çağlar, Mehmet Akif Güleç

**Affiliations:** ^1^Bağcılar Training & Research Hospital, Department of Orthopedics and Traumatology, Mimar Sinan Cad.6 Sok., Bağcılar, 34200 İstanbul, Turkey; ^2^Bağcılar Training & Research Hospital, Department of Pathology, Mimar Sinan Cad.6 Sok., Bağcılar, 34200 İstanbul, Turkey; ^3^Bağcılar Training & Research Hospital, Department of Radiology, Mimar Sinan Cad.6 Sok., Bağcılar, 34200 İstanbul, Turkey

## Abstract

Tenosynovial giant cell tumor is a locally aggressive tumor arising from the synovia of the fibrous tissue surrounding the joints, tendon sheaths, mucosal bursas, and tendons. Although it is often to be observed at the hand, localized form is very rare in the knee joint. In this case report, we aimed to present a very rare case of a surgically treated intra-articular giant cell tenosynovial tumor arising from the hoffa's infrapatellar fat pad of a 19-year-old male patient, by reviewing the literature. The patient we have treated with marginal excision was asymptomatic at the 14th month in the controls and recurrence was not detected.

## 1. Introduction

Tenosynovial giant cell tumor is a locally aggressive tumor arising from the synovia of the fibrous tissue surrounding the joints, tendon sheaths, mucosal bursas, and tendons [1–3]. Tenosynovial giant cell tumor, first described by Jaffe et al in 1941, is also known as pigmented villonodular synovitis [4]. There are localized and diffuse forms. Localized form is usually seen in the palmar region of hand and is rarely seen in the foot [1–8]. Tenosynovial localized giant cell tumor is very rare in the joint [1–3]. In this case report, we aimed to present a very rare case of a surgically treated intra-articular giant cell tenosynovial tumor arising from the tenosynovial tissue of the knee joint of a 19-year-old male patient, by reviewing the literature.

## 2. Case Report

**Figure 1:**
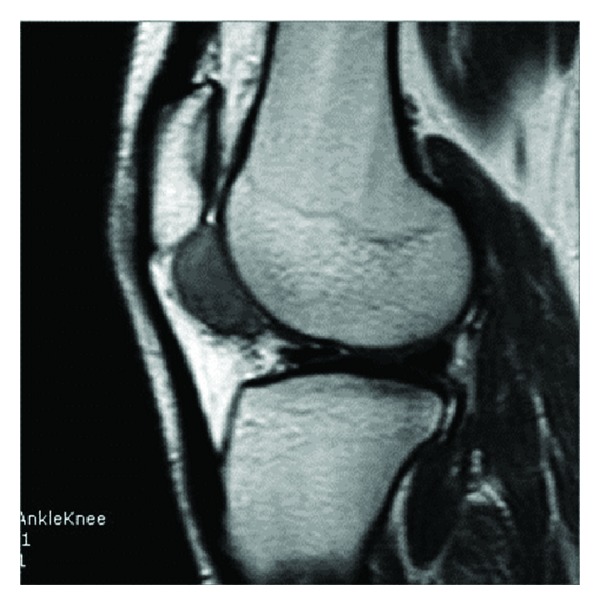
Hypointense lesion in infrapatellar region on T1-weighted MRI sequences.

**Figure 2:**
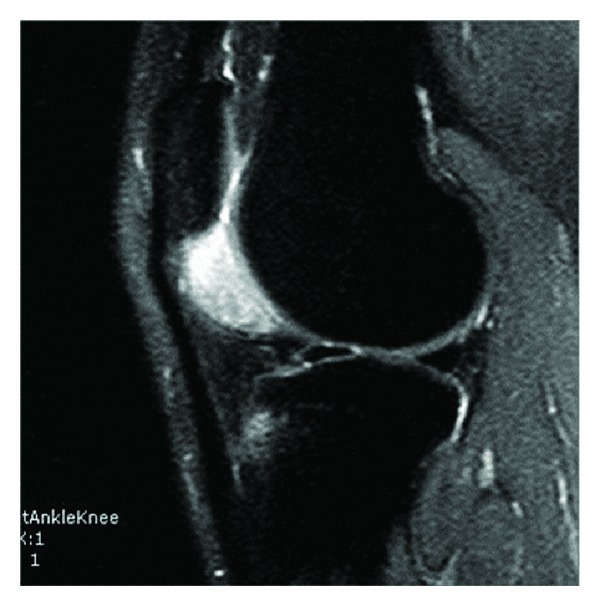
Hyperintense lesion in infrapatellar region on T2-weighted MRI sequences.

19-year-old male patient was admitted to our clinic with complaints of swelling and pain increasing with activity in his left knee. The patient had no pain at rest. On the examination of the patient there was a 2 × 3 cm in size swelling that appeared during flexion at the lateral side of the infrapatellar region of the left knee. Lachman, anterior drawer, posterior drawer, and medial and lateral stress tests were negative. Mc Murrey and Apley tests were also negative. The laboratory tests were normal. No abnormal findings were found at the X-ray. A heterogeneous, 3 × 2 cm sized, well-circumscribed lesion localized into the infrapatellar fat pad in the left knee was present at the MR-imaging. The lesion was hypointense on T1-weighted images and hyperintense on T2-weighted images (Figures 1 and 2).

Operation was planned because the lesion was symptomatic. Although the lesion was well-circumscribed, the lesion was heterogeneous, pathologies such as synovial sarcoma could be observed in this region, so, firstly, ultrasonography-guided needle aspiration biopsy was performed. Cytopathologically malignant cells were not seen and giant cell tumor of tendon sheath was reported. Thereupon marginal excision with open surgery was decided.

**Figure 3:**
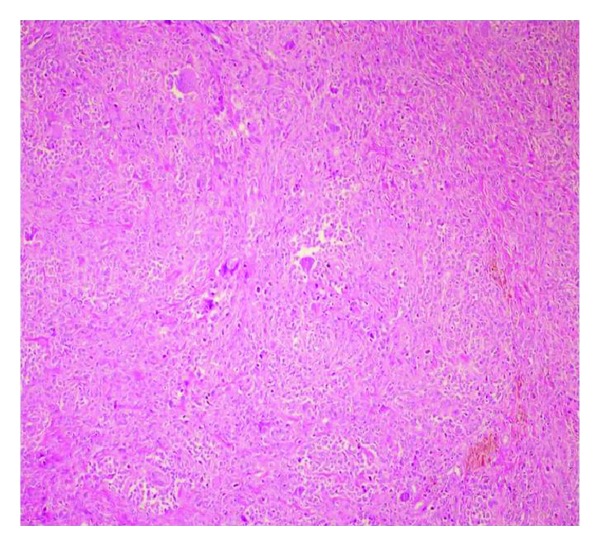
Multinucleated giant cells scattered on the floor containing collagenous stroma and round mononuclear cells (H.E. ×100).

Three cm longitudinal incision was made at the lateral side of patellar tendon of the left knee in the supine position under spinal anesthesia under tourniquet. After reaching the joint capsule, the lesion was reached by longitudinal arthrotomy. The lesion was excised marginally. Giant cell tenosynovial tumor was diagnosed at the histopathological examination (Figure 3).

Postoperative range of motion exercises were started the day after surgery. The patient was asymptomatic at the 14th month control and recurrence was not detected.

## 3. Discussion

Tenosynovial giant cell tumors originate from the synovial tissue of various regions of the body [1–8]. It is more common in women and the average age range is 30–50 [2–4]. Patients are usually presented with a painless, slowly growing mass. There are localized and diffuse forms. Although it is often to be observed at the hand, localized form is very rare in the knee joint. Plain radiographs are usually normal while MRI is very helpful in diagnosis. MRI shows well-circumscribed heterogeneous lesion, due to the of hemosiderin pigment content in the lesion [6]. Malignant tumors, such as synovial sarcoma and benign soft tissue tumors, should be kept in mind in differential diagnosis. Synovial sarcoma lesions usually create reaction and disorders in the adjacent bone. As in our case, benign tumors usually do not cause a reaction in the adjacent bone.

Marginal excision is sufficient in the treatment. Recurrence rate is between 10 and 20% [8]. In the literature, arthroscopic or open excision of intra-articular giant cell tumors has been reported. Although functional outcome is seen more advantageous at arthroscopic surgical treatment, intralesional removal of the tumor by fragmentation with the help of shaver creates doubt in terms of tumor surgery principles. Therefore we chose marginal excision of the mass with open surgery without shredding the lesion in our case. Recurrence rate is between 9 and 44% and is usually associated with inadequate excision [8]. Recurrence was not detected at the 14th month follow-up.

As a result although tenosynovial giant cell tumors localized in the knee joint are very rare; they should be kept in mind in the differential diagnosis. MRI is helpful in diagnosis and marginal excision is sufficient in the treatment.
